# 胸腺瘤的分期进展

**DOI:** 10.3779/j.issn.1009-3419.2011.02.13

**Published:** 2011-02-20

**Authors:** 艾则麦提·如斯旦木, 永清 王

**Affiliations:** 310003 杭州，浙江大学医学院附属第一医院胸外科 Department of Thoracic Surgery, First Affiliated Hospital of Zhejiang University School of Medicine, Hangzhou 310003, China

胸腺瘤（thymoma）是一种特殊类型的肿瘤，绝大多数位于前纵隔，少数可位于颈部、胸膜、肺门及肺实质内。发病率每年约0.05/10万人口，任何年龄均好发，可8个月-90岁不等，平均年龄53岁，男女发病率约相等^[1, 2]^。胸腺瘤的分期一直被认为是预测疗效和判断预后的重要变量，但由于它不同于其它器官的肿瘤，因此不能照搬TNM系统的分期方法。有关胸腺瘤分期的文献报道不多，至今仍在不断地更新其分期方法，现将这一内容综述如下，旨在帮助提高认识和规范治疗。

1978年，Berg等^[3]^最早推出胸腺瘤的分期标准，主要依据胸腺瘤有无包膜以及是否侵犯周围器官和胸腔内转移进行分期，此方法曾经得到广泛应用，虽然1979年Wilkins和Castleman^[4]^推荐了另一种胸腺瘤的分期标准（表 1），但大同小异。这两种分期方法存在的不足之处在于：①胸腺瘤在胸腔内实际转移的部位不明确；②对胸腺瘤侵袭范围的描述不完善；③Ⅲ期胸腺瘤的范围太广；④胸腺瘤的淋巴结转移和血行转移的特点未得到重视。1981年，日本学者Masaoka等提出了手术所见与显微镜所见相结合的分期系统，称之为胸腺瘤Masaoka分期（表 2）。该分期强调以下两点：①虽然肉眼观察肿瘤可能在包膜内，但镜下所见已超出胸腺包膜，分期应该有区别；②将具有恶性行为的胸腺瘤进一步细化。部分胸腺瘤病理可能无法证实胸腺瘤是否侵犯纵隔胸膜，但术中可以明确是否侵犯纵隔胸膜，因此要求手术者有描述，并依据实际情况进行分期；对于侵袭性的肿瘤则依据局部侵犯或广泛侵犯分为Ⅲ期和Ⅳa期，将远处转移的或任何纵隔淋巴结转移的胸腺瘤纳入Ⅳb期。

**1:** 胸腺瘤Berg分期和Wilkin and Castleman分期

Berg分期(1978)		Wilkins and Castleman分期(1979)	
Ⅰ期	有完整的包膜，包膜内生长	Ⅰ期	有完整的包膜，包膜内生长
Ⅱ期	包膜外生长，侵犯纵隔脂肪	Ⅱ期	包膜外生长，侵犯纵隔脂肪，或邻近胸膜，或心包
Ⅲ期	侵犯周围器官，胸腔内转移	Ⅲ期	侵犯周围器官，胸腔内转移

**2:** 胸腺瘤Masaoka分期

Ⅰ期	肿瘤局限在胸腺内，肉眼及镜下均无包膜浸润
Ⅱa期	肿瘤镜下超出胸腺包膜
Ⅱb期	侵犯邻近脂肪组织，但未侵犯至纵隔胸膜
Ⅲ期	肿瘤侵犯邻近组织或器官，包括心包、肺或大血管
Ⅳa期	肿瘤广泛侵犯胸膜和（或）心包
Ⅳb期	肿瘤扩散到远处器官

1991年法国多中心提出了GETT分期系统（Grouped’Etudes des Tumeurs Thymiques, GETT）^[5]^，此分期系统最重要的特点是根据分期推荐外科切除胸腺瘤的范围（表 3）。随着病例数量的积累，许多学者发现部分胸腺瘤在切除的纵隔脂肪组织中发现转移的淋巴结，并与预后有一定相关性，因此有必要对胸腺瘤提出TNM分期。Yamakawa和Masaoka等^[6]^率先在1991年推出了胸腺瘤的（Y-M）TNM分期（表 4），此分期的T完全运用了Masaoka分期方法，将淋巴结转移分为：N1指前纵隔淋巴结转移，N2指其他纵隔淋巴结（N1除外）的转移，N3指胸腔外淋巴结转移，M1指血行或远处转移。在Y-M胸腺瘤TNM分期中T1N0M0属于Ⅰ期，T2N0M0属于Ⅱ期，T3N0M0属于Ⅲ期，T4N0M0属于Ⅳa期，任何T、N1-N3、M0或任何T、任何N、M1均属于Ⅳb期。TNM分期认为淋巴结转移和血行或远处转移一样，预后较差。

**3:** 法国胸腺瘤GETT分期系统（1991年）

Ⅰa期	包膜完整，可完全切除
Ⅰb期	肉眼包膜完整，可完全切除，但怀疑胸腺瘤与纵隔胸膜粘连和潜在包膜侵犯可能
Ⅱ期	侵袭性胸腺瘤，可完整切除
Ⅲa期	侵袭性胸腺瘤，可次全切除（部分切除）
Ⅲb期	侵袭性胸腺瘤，仅活检
Ⅳa期	锁骨上淋巴结转移，或远处胸膜种植
Ⅳb期	远处转移

**4:** 胸腺瘤Y-M TNM分期（1991年）

TⅠ	瘤局限在胸腺内，肉眼及镜下均无包膜浸润
T2	肿瘤镜下超出胸腺包膜，侵犯邻近脂肪组织或纵隔胸膜
T3	肿瘤侵犯邻近组织或器官，包括心包、肺或大血管
T4	肿瘤广泛侵犯胸膜和（或）心包
N0	无淋巴结转移
N1	前纵隔淋巴结转移
N2	胸腔内淋巴结转移（NI除外）
N3	胸腔外淋巴结转移
M0	无血行或远处转移
M1	有血行或远处转移

2004年，世界卫生组织（World Health Organization, WHO）发表了胸腺瘤的WHO分期^[7]^，它与Masaoka分期的不同点是关于N1的划分，WHO分期将N1划分为Ⅲ期，而Masaoka分期中，N1则属于Ⅳb期（表 5）。在WHO胸腺瘤TNM分期中，T1N0M0属于Ⅰ期。T2N0M0属于Ⅱ期。T1N1M0、T2NIM0、T3N0M0、T3N1M0均属于Ⅲ期、T4任何NM0、任何TN2（或N3）M0、任何T任何NM1均属于Ⅳb期。

**5:** 胸腺瘤WHO TNM分期（2004年）

T1	包膜完整
T2	肿瘤浸润包膜外结缔组织
T3	肿瘤浸润邻近组织器官。如，心包、纵隔胸膜、胸壁、大血管及肺
T4	肿瘤广泛侵犯胸膜和（或）心包
N0	无淋巴结转移
N1	前纵隔淋巴结转移
N2	N1+胸内淋巴结转移
N3	前斜角肌或锁骨上淋巴结转移
M0	无远处转移
M1	有远处转移

采用TNM分期的方法逐渐得到广大学者的认同，2005年Bedini等^[8]^发表了最新胸腺瘤TNM分期系统，称之为Istituto Nazionale Tumori（INT）（表 6），认为此分期方法更具有临床应用价值。但究竟如何仍需要通过多中心合作来证实是否合理。

**6:** Istituto Nazionale Tumori（INT分类2005年)

T1	肉眼包膜完整，镜检无包膜浸润
T2	镜下或肉眼肿瘤侵润包膜或侵犯周围脂肪组织或正常胸腺
T3	直接侵犯纵隔胸膜和/或前心包
T4	T3+直接侵犯周围器官，如胸骨，大血管，肺等
N0	无淋巴结转移
N1	前纵隔淋巴结转移
N2	N1+胸内淋巴结转移
N3	前斜角肌或锁骨上淋巴结转移
M0	无远处转移
M1a	超出T4提到的部位，扩散到纵隔胸膜和心包
M1b	远处转移，或转移至N系统未提到的其他部位的淋巴结
R0	无残留灶
R1	镜下残留灶
R2a	部分切除肿块（> 80%)后局部肉眼残留灶
R2b	残留灶的其他特点

此分期系统中Ⅰ期（局部早期病灶）：T1-2、N0M0，Ⅱ期（局部晚期病灶）：T3-4、N0M0，或任何T、N1-2M0，Ⅲ期（系统性疾病）：任何T、N3M0，或任何T任何N、M。虽然恶性肿瘤的TNM分期中N对肿瘤的分期起重要作用，但胸腺瘤的淋巴结转移非常罕见，Kondo等^[9]^总结了1, 064例胸腺瘤中，只有16例（1.8%）有淋巴结转移。故胸腺瘤淋巴结转移的生物学行为仍需要再进一步研究。为了便于比较，我们将上述胸腺瘤的TNM分期与5年生存率的关系列表如下（表 7）。

**7:** 不同胸腺瘤的TNM分期与5年生存率的比较

分期	M-Y分期	5年生存率	WHO分期	5年生存率	INT分期	5年生存率
1期	T1N0M0	94%	T1N0M0	不详	T1-2, N0M0	99%
Ⅱ期	T2N0M0	90%	T2N0M0	不详	T3-4, N0M0 Tany, N1-2M0	47%
Ⅲ期	T3N0M0	72%	T1N1M0 T2NIM0 T3N0/IM0	不详	Tany N3M0 Tany Nany M1	12%
Ⅳ期	Ⅳa; T4N0M0	56%	T4NanyM0 TanyN2-3M0 TanyNanyM1	不详	无	无
	Ⅳb: TanyN1-3M0, TanyNanyMl	78%				

胸腺瘤作为一种具有良性细胞学特征恶性生物学行为的肿瘤，其预后好于其他器官的恶性肿瘤，应采取积极的治疗，其中手术切除仍是主要治疗手段。对于侵袭性胸腺瘤或有淋巴结转移或远处转移的患者，应辅助放化疗。胸腺瘤的另一个特点还具有多种合并症（表 8），根据国外流行病学资料显示，10%-20%的重症肌无力患者合并有胸腺瘤，而20%-25%的胸腺瘤患者合并重症肌无力^[10, 11]^，约10%-15%胸腺瘤患者合并副肿瘤综合症^[12]^，其治疗方法也各不相同。因此胸腺瘤是一种非常特殊类型的肿瘤，如何寻找一种能兼顾合理治疗的分期方法，仍然需要不断地探索。

**8:** 胸腺瘤合并的副肿瘤综合症

神经肌肉综合征：重症肌无力、Eaten-Lambert综合征、强直性肌营养不良症、边缘性脑病、僵硬人症候群
胃肠道疾病：慢性溃疡性结肠炎、局限性肠炎
胶原蛋白和自身免疫疾病：系统性红斑狼疮、结节病、类风湿性关节炎、多发性肌炎、皮肤肌炎、心包炎、干燥综合症、雷诺氏病、甲状腺炎
皮肤疾病：天疱疹、脱发、慢性念珠菌感染
内分泌系统疾病：Cushing综合征、甲状腺功能低下、Addison病、肥大性骨关节病
泌尿系统疾病：肾病、微小病变肾病
造血系统疾病：红细胞再生障碍性贫血、红细胞发育不全、恶性贫血、红细胞增多症、粒细胞缺乏症、多发性骨髓瘤、溶血性贫血、急性白血病、T-淋巴细胞增多症
免疫缺陷综合征：低丙种球蛋白血症、T-淋巴细胞缺乏症
